# Unveiling the role of melatonin‐related gene *CSNK1D* in osteoclastogenesis and its implications for osteoporosis treatment

**DOI:** 10.1113/EP092189

**Published:** 2024-11-29

**Authors:** Jiewen Zhang, Shaobo Wu, Fangze Xing, Ning Kong, Yiwei Zhao, Xudong Duan, Yiyang Li, Kunzheng Wang, Run Tian, Pei Yang

**Affiliations:** ^1^ Joint & Ankle Section The Second Affiliated Hospital of Xi'an Jiaotong University Xi'an China; ^2^ Department of Spinal Surgery, Honghui Hospital Xi'an Jiaotong University Xi'an China

**Keywords:** circadian rhythm, *CSNK1D*, melatonin, osteoclast, osteoporosis

## Abstract

Osteoporosis (OP) is a prevalent bone disease characterized by reduced bone density and quality, increasing fragility and fracture risk. Osteoclast (OC) activity and circadian rhythm play a role in the pathogenesis of OP. Melatonin is a circadian regulator that affects bone metabolism, but its molecular mechanism has not been studied in detail. This study aimed to identify the relationship between melatonin‐related genes and OP through bioinformatics methods and to verify it experimentally.We analysed microarray data from the GSE35959 dataset, identifying differentially expressed genes in OP patients. Circadian rhythm‐related genes and melatonin‐related genes intersect with these differentially expressed genes, highlighting that *CSNK1D* is a central gene. Functional enrichment, correlation and protein–protein interaction analyses were conducted. Experimental validation involved in vitro differentiation assays using RAW264.7 cells and in vivo studies with an ovariectomy‐induced rat model of OP to evaluate the role of *CSNK1D* in osteoclastogenesis to verify its effect on OP. Differential expression analysis revealed 272 significant genes, with *CSNK1D* identified as central to the circadian rhythm and to melatonin and OP interplay. Functional analyses showed involvement of *CSNK1D* in OC differentiation and inflammatory pathways. in vitro experiments confirmed *CSNK1D* upregulation during OC differentiation, and small interfering RNA‐mediated knockdown reduced OC marker expression and TRAP^+^ cell formation. in vivo, *CSNK1D* expression is associated with bone loss in OP rats. Melatonin‐related *CSNK1D* promotes OC differentiation and promotes the development of OP. These findings suggest *CSNK1D* as a potential therapeutic target for OP, offering insights into new treatment strategies integrating circadian rhythm regulation.

## Abstract (highlights)

- **What is the central question of this study?**
  What is the relationship between melatonin‐related genes and osteoporosis?
- **What is the main finding and its importance?**
  We found that the melatonin‐related gene *CSNK1D* plays an important role in the occurrence of osteoporosis. These findings suggest that *CSNK1D* is a key regulator of osteoporosis pathogenesis, linking circadian rhythms and melatonin signalling to osteoclasts, osteoblasts and bone metabolism. By targeting *CSNK1D*, it might be possible to develop therapies that more effectively prevent or treat osteoporosis, reducing fracture risk and improving bone health in affected individuals.

## 1 INTRODUCTION

Osteoporosis (OP) is a prevalent bone disease characterized by a significant reduction in bone density and quality, resulting in increased bone fragility and a higher susceptibility to fractures (Black & Rosen, 2016; McClung, 1999; Siris et al., 2001). This condition has become a major global public health challenge, especially with the ageing population, leading to a notable rise in incidence (Camacho et al., 2020; Kanis et al., 2019). In addition, owing to the dramatic drop in sex hormone levels after menopause, women are at a much higher risk of OP than men, which is manifested further by a significant increase in the prevalence of OP‐related fractures (52%) with age, especially in women >80 years of age (Kanis, 1994; Neer et al., 2001). The onset of OP involves intricate imbalances in bone metabolism, with the abnormal activity of osteoclasts (OCs) playing a pivotal role in bone loss (Rachner et al., 2011). Osteoclasts, responsible for bone resorption, exacerbate the progression of OP by breaking down bone tissue (Boyle et al., 2003). The Wnt/β‐catenin signalling pathway is integral to the regulation of bone metabolism (Huybrechts et al., 2020). It promotes the formation and activity of osteoblasts, the cells responsible for bone formation, while concurrently inhibiting the activity of OCs (Liu et al., 2007). This dual action helps to maintain a balance in bone mass and is crucial in preventing excessive bone loss (Hadjidakis & Androulakis, 2006). Osteoporosis often leads to fractures in crucial areas such as the hip, spine and wrist (Coughlan & Dockery, 2014). These fractures not only drastically diminish the quality of life for affected individuals but also impose substantial healthcare costs and societal burdens (Clynes et al., 2020). By understanding the underlying pathological mechanisms of OP, including the roles of OCs, more effective therapeutic strategies can be developed.

The circadian rhythm, an intrinsic 24 h cycle regulating various physiological processes, plays a crucial role in maintaining bone health (Swanson et al., 2018). This rhythm is influenced by external cues, such as light and dark, and disruptions, such as shift work, jet lag and irregular sleep patterns, can adversely affect bone density and metabolism (Wirz‐Justice et al., 2021). Recent studies indicate that circadian rhythm disruption not only accelerates bone loss but also impairs bone formation, highlighting the importance of a well‐regulated circadian cycle in bone homeostasis (Juliana et al., 2023). Melatonin, a hormone primarily produced by the pineal gland at night, is integral to circadian rhythm regulation (Vasey et al., 2021). Its production decreases with age, immobility and menopause, factors contributing to OP risk (MacDonald et al., 2021). Melatonin receptors are present in bone cells, suggesting that melatonin has a significant role in bone physiology (Amstrup et al., 2013). Studies indicate that melatonin enhances bone formation by promoting osteoblast activity and inhibiting osteoclastogenesis, the process by which OCs break down bone tissue (Cardinali et al., 2003). The bone‐protective effects of melatonin are mediated through various signalling pathways, including ERK1/2 and NF‐κB, which are crucial for OC differentiation and function (Ping et al., 2017). Research has shown that administration of melatonin can restore normal nocturnal melatonin levels, thereby offering protective effects against bone loss and fractures (Zheng et al., 2022). Despite these findings, the precise mechanisms by which melatonin and circadian rhythms influence OC activity and overall bone metabolism remain incompletely understood.

Osteoclasts, large multinucleated cells derived from haematopoietic precursors of the monocyte–macrophage lineage, are essential for the maintenance of bone integrity through their specialized function in resorbing mineralized bone matrix (Roodman, 1996; Teitelbaum & Ross, 2003; Zaidi et al., 1993). This process is finely tuned by a complex interplay of signalling pathways, among which the canonical Wnt pathway plays a critical role (Liu et al., 2008). Activation of OCs is initiated by the binding of the receptor activator of nuclear factor kappa‐Β ligand (RANKL) to its receptor, RANK, on OC precursor cells (Boyce, 2013; Krishnacoumar et al., 2024). This interaction triggers downstream signalling cascades that culminate in the activation of transcription factors essential for OC differentiation and activity, notably the nuclear factor of activated T cells cytoplasmic 1 (NFATc1) (Komatsu et al., 2024; Zeng et al., 2016). Dysregulation of the RANKL/RANK/osteoprotegerin (OPG) axis, often characterized by elevated RANKL expression or impaired OPG function, disrupts this balance and promotes unchecked osteoclastic activity, ultimately leading to the characteristic bone loss observed in OP (Boyce & Xing, 2007, 2008; Zhao et al., 2024). In addition, canonical Wnt signalling inhibits OC differentiation by antagonizing NFATc1 activation through various mechanisms, including the stabilization of β‐catenin and subsequent inhibition of NFATc1 transcriptional activity (Weivoda et al., 2016). Conversely, non‐canonical Wnt pathways, such as the Wnt5a pathway, can stimulate OC differentiation and function, particularly in inflammatory or pathological conditions (Maeda et al., 2012). Alterations in Wnt signalling components, such as mutations in low‐density lipoprotein receptor‐related protein 5, have been implicated in various skeletal disorders, highlighting the intricate dual role of Wnt pathways in modulating OC activity and bone remodelling dynamics in OP (Yue et al., 2022). Osteoblasts, derived from mesenchymal stem cells, are integral in maintaining bone integrity by synthesizing and mineralizing new bone matrix (Titorencu et al., 2014). In OP, osteoblast activity is diminished, contributing to further bone loss (Manolagas & Jilka, 1995). Previous research has highlighted the importance of circadian regulation in osteoblast activity, showing that disruptions in circadian rhythms can lead to reduced osteoblast function and impaired bone formation (Takarada et al., 2017).

In this study, we obtained OP sequencing data from the gene expression omnibus (GEO) database. We identified genes associated with OP through differential expression analysis. These differential expression genes were then intersected with circadian rhythm‐related genes to pinpoint central genes. To refine our focus, we also analysed the correlation between these central genes and angiogenesis, chemokines and inflammatory factors. Among the central genes, we identified one related to melatonin and investigated its function in detail. Our experimental approach includes in vitro and in vivo validations to elucidate the role of this melatonin‐related gene in OP. These experiments aim to confirm the influence of the gene on bone metabolism and its potential as a therapeutic target. We hope to uncover new molecular mechanisms underlying OP and develop innovative therapeutic strategies by integrating bioinformatics tools with experimental biology.

## 2 MATERIALS AND METHODS

### 2.1 Ethical approval

All animal procedures were approved by the Institutional Animal Care and Use Committee of Xi'an Jiaotong University (No. 2021‐1567). Twelve 12‐week‐old female Sprague–Dawley rats, each weighing ∼300 ± 20 g, were obtained from the Animal Center of Xi'an Jiaotong University School of Medicine. The rats were housed, three per cage, in specific pathogen‐free conditions, maintained at a temperature of 22°C–24°C with 50%–60% humidity, and subjected to a 12 h–12 h light–dark cycle, with the lights turned on at 07.00 h and turned off at 19.00 h. They had unrestricted access to water and a standard laboratory rodent diet. After a 1 week acclimation period, the rats were divided randomly into two groups: a normal control (NC) group and an ovariectomy group. At the designated time point, OP was induced in the ovariectomy group via bilateral ovariectomy, whereas the NC group underwent a sham operation. Ten weeks postsurgery, the rats were killed using an overdose of pentobarbitone sodium (150 mg/kg, i.p.; Shanghai Xinya Pharmaceutical Co., Ltd, Shanghai, China). As soon as death was confirmed, both femurs were removed, processed, and either stored at −80°C or fixed in 4% paraformaldehyde (Servicebio, Wuhan, China) for subsequent analyses.

### 2.2 Data acquisition and preprocessing

Microarray data from the GSE35959 dataset, which includes samples from five OP patients (aged 79–94 years) and nine control subjects (aged 42–67 and 79–89 years), was downloaded from the GEO database. Differentially expressed genes (DEGs) were identified with thresholds set at |Log2FC| > 1 and a *P*‐value of < .05. Then, we intersected with 248 circadian rhythm‐related genes by Venn diagram to identify circadian‐related DEGs associated with OP. Four genes were pinpointed as central to the circadian rhythm and OP interplay. Finally, the four DEGs were intersected with circadian rhythm‐related genes and melatonin‐related genes to identify the final central gene. Molecular Signatures Database (MSigDB; https://www.gsea‐msigdb.org/gsea/msigdb) is a resource of annotated gene sets for use with GSEA software, divided into Human and Mouse collections. A total of 248 circadian rhythm‐related genes were extracted from 13 gene sets in MSigDB.

### 2.3 Enrichment analysis

Gene Ontology (GO) enrichment analysis, covering biological processes (BP), cellular components (CC) and molecular functions (MF), along with Kyoto Encyclopedia of Genes and Genomes (KEGG) pathway analysis, were conducted on the identified hub genes. A *P*‐value threshold of <0.05 was used to determine significant enrichment. This analysis facilitated the understanding of biological functions and pathways associated with the identified melatonin‐related central genes. Then, GO and KEGG were used to explore the function of the final central gene.

### 2.4 Correlation analysis

Correlation analyses were conducted to explore the relationships between the identified hub genes and key factors involved in angiogenesis, chemokine activity and inflammation. Statistical tools were used to determine the strength and significance of these correlations (*P* < 0.05).

### 2.5 Protein−protein interaction network analysis

The protein−protein interaction (PPI) network for the central genes was constructed using the STRING database. with an interaction score threshold of >0.4. The resulting network was visualized and analysed using Cytoscape software (v.3.9.0) to identify key interaction modules and pathways involved in OP.

### 2.6 Target gene transcription factor prediction

The KnockTF database (http://www.licpathway.net/KnockTF/index.html) is based on the T(co)F knockdown/related knockout‐related human gene expression data. The ConTra v.3 database allows easy visualization and exploration of predicted transcription factor binding sites (TFBSs) in any genomic region around coding or non‐coding genes.

### 2.7 Reagents and antibodies

RAW264.7 cells were purchased from Wuhan Prosa Life Science Co., Ltd. RANKL proteins were purchased from PeproTech Inc. The TRAP staining kit was obtained from Wako (Japan). Cell culture reagents, including minimal Eagle's medium (MEM), penicillin–streptomycin solution and fetal bovine serum (FBS), were provided by Thermo Fisher Scientific Inc. Various antibodies, such as CSNK1D (Proteintech, catalogue number 14388‐1‐AP), Matrix metalloproteinase‐9 (MMP9) and β‐actin (Cell Signaling Technology, catalogue numbers 3852 and 4967), NFATc1 and c‐Fos (Santa Cruz Biotechnology, catalogue numbers sc‐7294 and sc‐166940), Runt‐related transcription factor 2 (RUNX2) and osteopomtin (OPN, Abcam, catalogue numbers ab236639 and ab283656) were sourced from the respective companies. Dimethyl sulfoxide (DMSO) was purchased from Sigma‐Aldrich.

### 2.8 Cell culture and osteoclast differentiation assays

RAW264.7 cells were purchased from Wuhan Prosa Life Science Co., Ltd. Upon receipt, cells were immediately thawed and cultured in MEM supplemented with 10% FBS, 1% penicillin–streptomycin and 2 mM l‐glutamine. The cells were maintained in a humidified incubator at 37°C with 5% CO_2_. The culture medium was replaced every 2–3 days to ensure optimal growth conditions. Once the RAW264.7 cells reached ∼80%–90% confluency, they were passaged. Cells were then seeded at a density of 1 × 10^4^ cells/cm^2^ in six‐well plates for differentiation experiments. To induce differentiation into OCs, RAW264.7 cells were treated with 100 ng/mL RANKL. The induction medium consisted of MEM supplemented with 10% FBS, 1% penicillin–streptomycin and 50 ng/mL RANKL. The cells were cultured for 4 days, and the differentiation medium was refreshed every 2 days to maintain the RANKL concentration.

### 2.9 Osteoblast culture and differentiation induction

MC3T3‐E1 pre‐osteoblasts (ATCC, RIKEN BRC, Tsukuba, Japan) were cultured in α‐MEM (Thermo Fisher Scientific Inc.) supplemented with 10% FBS and 1% penicillin–streptomycin. Cells were incubated at 37°C with 5% CO₂. For osteoblast differentiation, cells were seeded into six‐well plates at a density of 2 × 10^5^ cells per well. Osteogenic differentiation was induced by supplementing the culture medium with 50 µg/mL ascorbic acid (Sigma‐Aldrich) and 10 mM β‐glycerophosphate (Sigma‐Aldrich). Cells were cultured in differentiation conditions for ≤7 days, and the medium was changed every 2–3 days. At specific time points (day 0 and day 7), cells were collected for western blot analysis to evaluate the expression levels of RUNX2, OPN and CSNK1D.

### 2.10 Tartrate‐resistant acid phosphatase staining and F‐actin fluorescence staining

After 4 days of RANKL induction, the cells were fixed with 4% paraformaldehyde for 15 min at room temperature. Following fixation, cells were rinsed with PBS and subjected to tartrate‐resistant acid phosphatase (TRAP) staining. For TRAP staining, cells were incubated with a TRAP staining solution (Sigma‐Aldrich) prepared according to the manufacturer's instructions, at 37°C for 1 h. TRAP^+^ multinucleated cells were identified as mature OCs under a light microscope.

For F‐actin fluorescence staining, cells were permeabilized with 0.1% Triton X‐100 for 5 min, then incubated with Phalloidin‐iFluor 488 (Abcam, Shanghai, China) at room temperature for 30 min. After washing with PBS, nuclei were counterstained with 4′,6‐diamidino‐2‐phenylindole (DAPI; Sigma‐Aldrich) for 5 min. Stained cells were observed under a fluorescence microscope to assess F‐actin ring formation, characteristic of mature OCs.

### 2.11 Fluorescent staining of the central gene

Cells were permeabilized with 0.1% Triton X‐100 for 5 min, then incubated with Phalloidin‐iFluor 488 (Abcam, Shanghai, China) at room temperature for 30 min. After permeabilization, cells were blocked with 5% bovine serum albumin (Sigma‐Aldrich) in PBS for 1 h at room temperature. The cells were then incubated with a primary antibody specific to the central gene, *CSNK1D* (diluted in PBS with 1% bovine serum albumin), overnight at 4°C. After washing with PBS, cells were incubated with an appropriate fluorescently labelled secondary antibody for 1 h at room temperature, protected from light. After a final wash with PBS, the nuclei were counterstained with DAPI (Sigma‐Aldrich) for 5 min. Fluorescent images were captured using a fluorescence microscope to visualize the localization and expression of the central gene.

### 2.12 Western blot analysis

Protein extracts from cells were prepared using RIPA buffer supplemented with protease and phosphatase inhibitors. The protein concentration was determined using the Bicinchoninic acid (BCA) protein assay kit (Thermo Fisher Scientific). Equal amounts of protein were resolved by SDS‐PAGE and transferred onto polyvinylidene difluoride membranes. Membranes were blocked with 5% non‐fat dry milk in TBST (Tris‐buffered saline with 0.1% Tween 20) for 1 h at room temperature, followed by overnight incubation at 4°C with primary antibodies against OC markers (MMP9, NFATc1 and c‐Fos), osteoblast markers (RUNX2 and OPN) and the central gene, *CSNK1D*. After washing, membranes were incubated with horseradish peroxidase‐conjugated secondary antibodies for 1 h at room temperature. Protein bands were detected using an enhanced chemiluminescence (ECL) detection system (Bio‐Rad). The expression of β‐actin was used as a loading control.

### 2.13 Small interfering RNA‐mediated gene silencing

Small interfering RNA (siRNA) specific to the central gene was transfected into RAW264.7 cells using a suitable transfection reagent according to the manufacturer's protocol (Thermo Fisher Scientific Inc.). A non‐targeting siRNA was used as a control. Post‐transfection, cells were induced to differentiate into OCs using RANKL for 4 days.

### 2.14 Micro‐CT detection

After fixing rat femur samples in 4% paraformaldehyde for 3 days, micro‐CT scanning was performed on the distal femur. VG Studio Max 3.0 software was used for three‐dimensional reconstruction, and quantitative measures of the bone volume fraction, trabecular thickness and the number of trabeculae were obtained to evaluate bone mass and microstructure.

### 2.15 Tissue immunofluorescence staining

For the immunofluorescence analysis, femoral head sections were harvested from the ovariectomy group and the NC group. The bone tissues were fixed in 4% paraformaldehyde, decalcified with 10% EDTA at 4°C for 4 weeks, and embedded in paraffin. Sections (5 µm thick) were deparaffinized and subjected to antigen retrieval using citrate buffer. After blocking with 5% bovine serum albumin for 30 min, the sections were incubated overnight at 4°C with primary antibodies against CSNK1D (1:100) and OPN (1:100). The next day, the sections were washed and incubated with Alexa Fluor‐conjugated secondary antibodies (1:500) for 1 h at room temperature in the dark. Nuclei were counterstained with DAPI. Immunofluorescence signals were visualized using a fluorescence microscope, and images were captured. Quantification of fluorescence intensity was performed using ImageJ software.

### 2.16 Statistical analyses

Statistical analyses were conducted using GraphPad Prism 8.3 (GraphPad Software, San Diego, CA, USA). Each experiment was replicated at least three times. Data are presented as the mean ± SD. Student's unpaired *t*‐test was used for comparing two groups. Statistical significance was defined as *P* < 0.05.

## 3 RESULTS

### 3.1 Identification of differentially expressed circadian rhythm‐related genes

**FIGURE 1:**
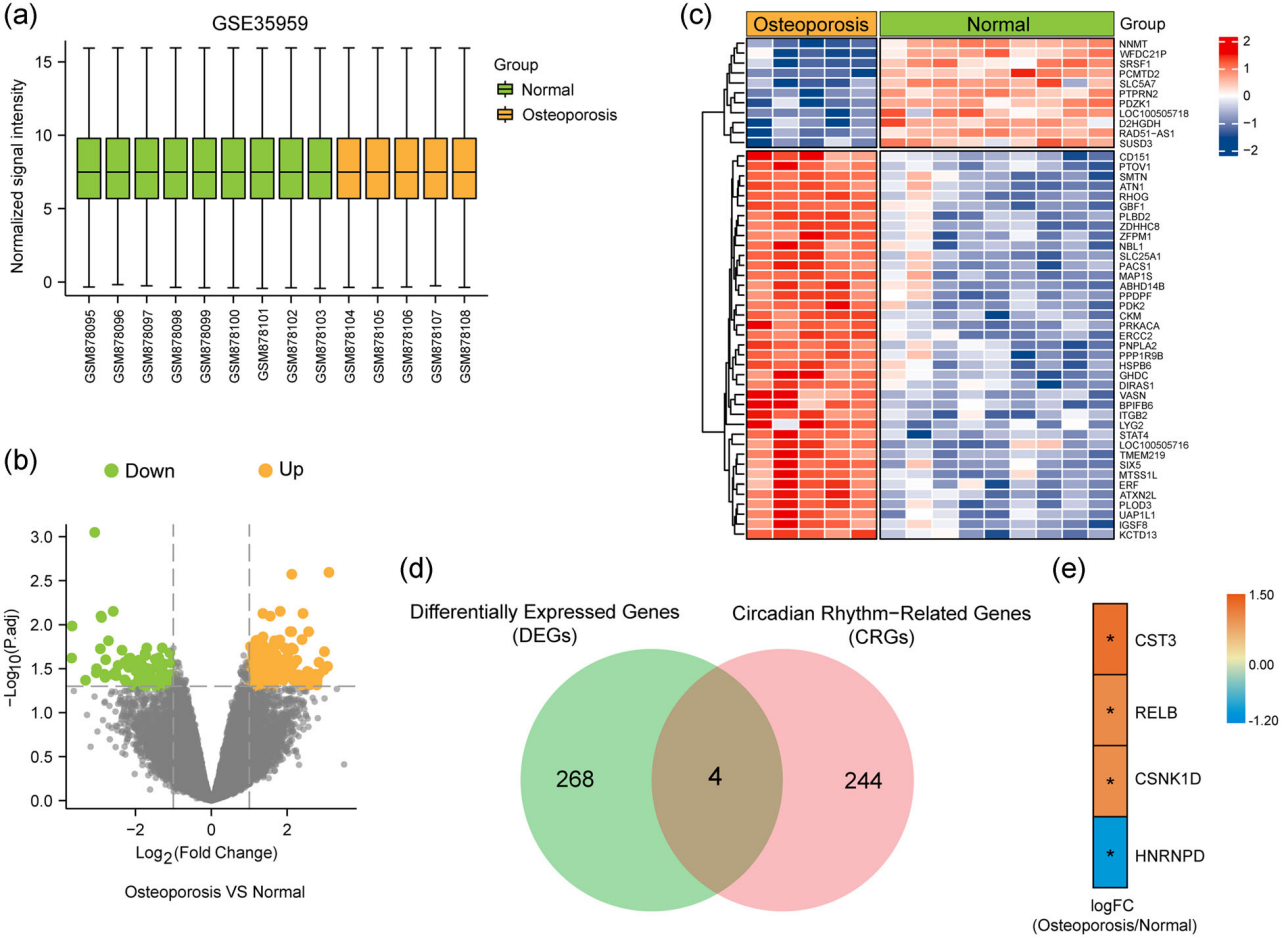
Identification of differentially expressed circadian rhythm‐related genes. (a) Homogenization of the dataset samples. (b) Volcano plot of differential gene analysis. (c) Heat map plot of the differential gene analysis. (d) Intersection of differential genes and genes related to circadian rhythm. (e) Heat map of expression patterns of four hub genes.

Analysis of GSE35959 data from nine normal individuals and five OP patients revealed good sample homogeneity (Figure 1a). Differential expression analysis identified 272 genes with significant differences between the groups, illustrated in a volcano plot (Figure 1b) and a heat map plot (Figure 1c). Using a curated list of 248 circadian rhythm‐related genes from MSigDB, we identified four hub genes crucial in OP: *CST3*, *RELB*, *HNRNPD* and *CSNK1D* (Figure 1d). Their expression patterns across groups were visualized using a heat map (Figure 1e).

### 3.2 Functional enrichment analysis of four hub genes

**FIGURE 2:**
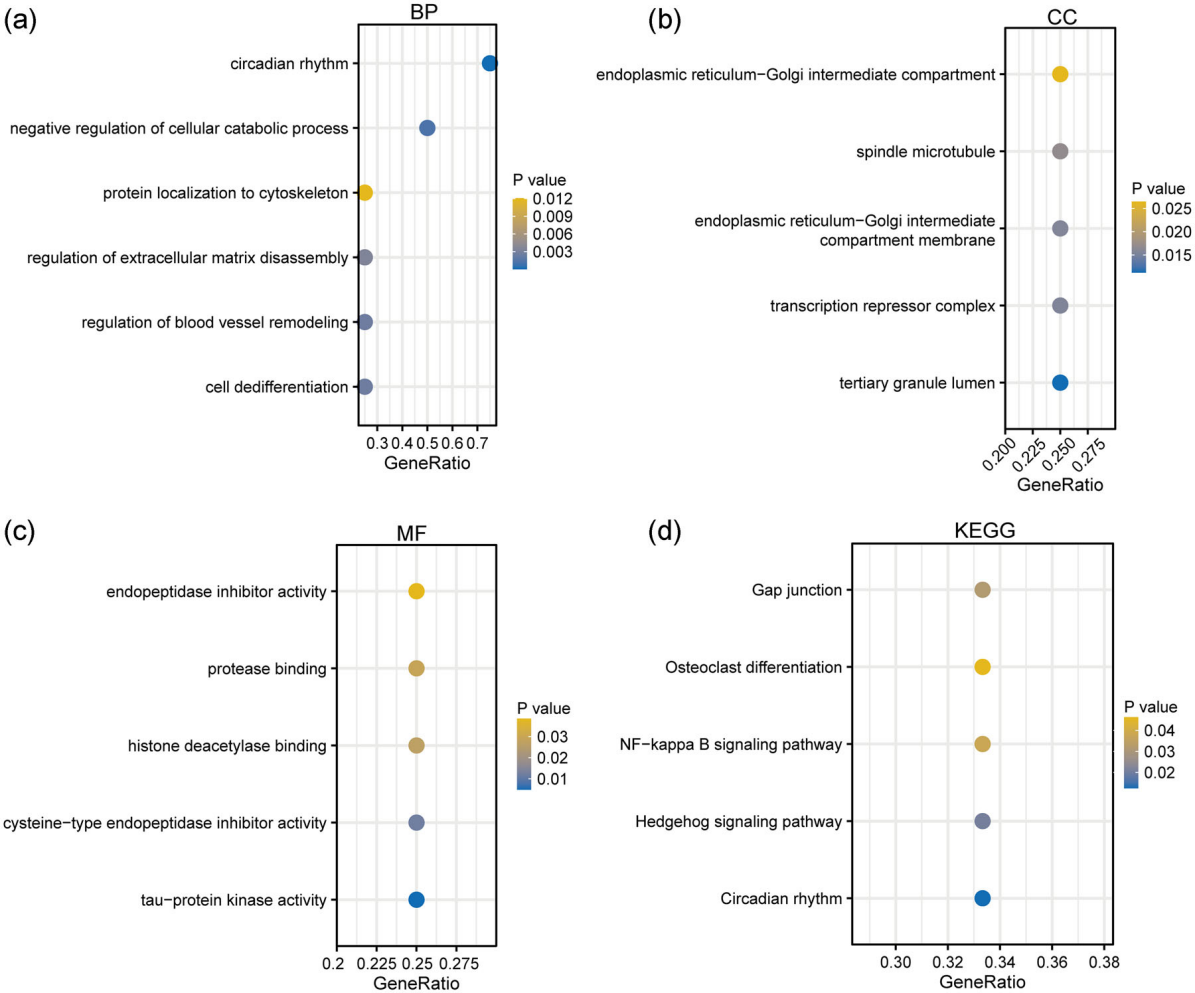
Functional enrichment analysis of four hub genes. (a) Biological process analysis. (b) Cellular component analysis. (c) Molecular function analysis. (d) KEGG pathway analysis. BP, Biological Process; CC, Cellular Component; MF, Molecular Function; KEGG, Kyoto Encyclopedia of Genes and Genomes.

Functional analysis highlighted the diverse roles of the four key hub genes. They were implicated in biological processes including circadian rhythm regulation, cellular process modulation and cell differentiation control (Figure 2a). In terms of cellular components, they are associated with specific cellular membranes and regulatory complexes (Figure 2b). Molecular functions included activities such as kinase function and protease binding (Figure 2c). KEGG pathway analysis revealed their involvement in pathways such as gap junction, OC differentiation and NF‐κB signalling (Figure 2d).

### 3.3 Correlation analysis of hub genes

**FIGURE 3:**
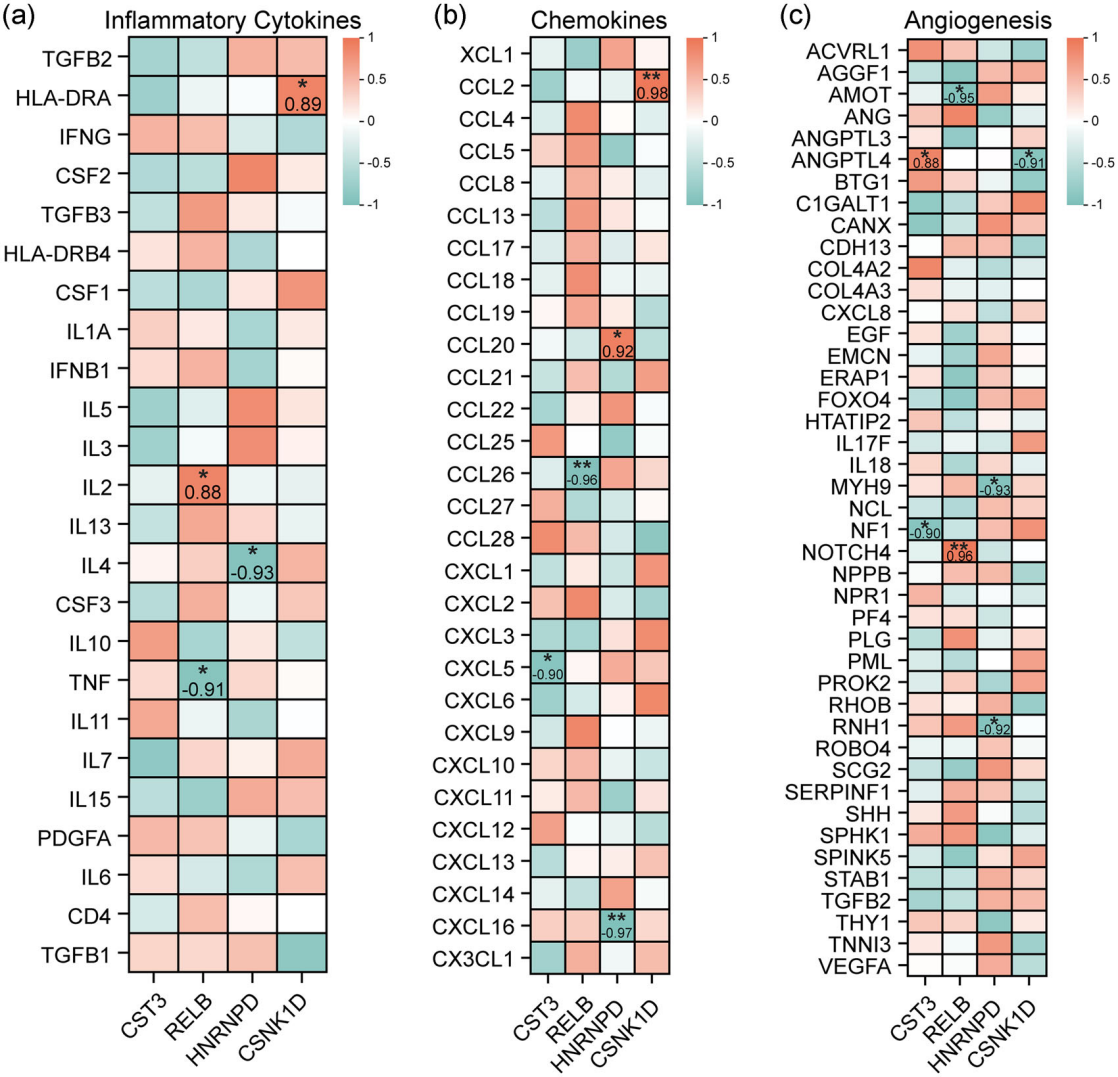
Correlation analysis of hub genes. (a) Heat map of correlation analysis between hub genes and inflammatory factors. (b) Heat map of correlation analysis between hub genes and chemokines. (c) Heat map of correlation analysis between hub genes and angiogenic factors.

Correlation analysis revealed significant relationships between the four key hub genes and inflammatory cytokines. For instance, *RELB* showed a strong negative correlation with *TNF* (correlation = −0.91, *P* = 0.03) and a positive correlation with *IL‐2* (correlation = 0.88, *P* = 0.02) (Figure 3a). *HNRNPD* exhibited a significant negative correlation with *IL‐4* (correlation = −0.93, *P* = 0.02), and *CSNK1D* correlated positively with *HLA‐DRA* (correlation = 0.89, *P* = 0.03). In addition, heat maps illustrate correlations of these key hub genes with chemokines (Figure 3b) and angiogenesis‐related factors (Figure 3c).

### 3.4 Identification of the key gene, *CSNK1D*, and correlation analysis

**FIGURE 4:**
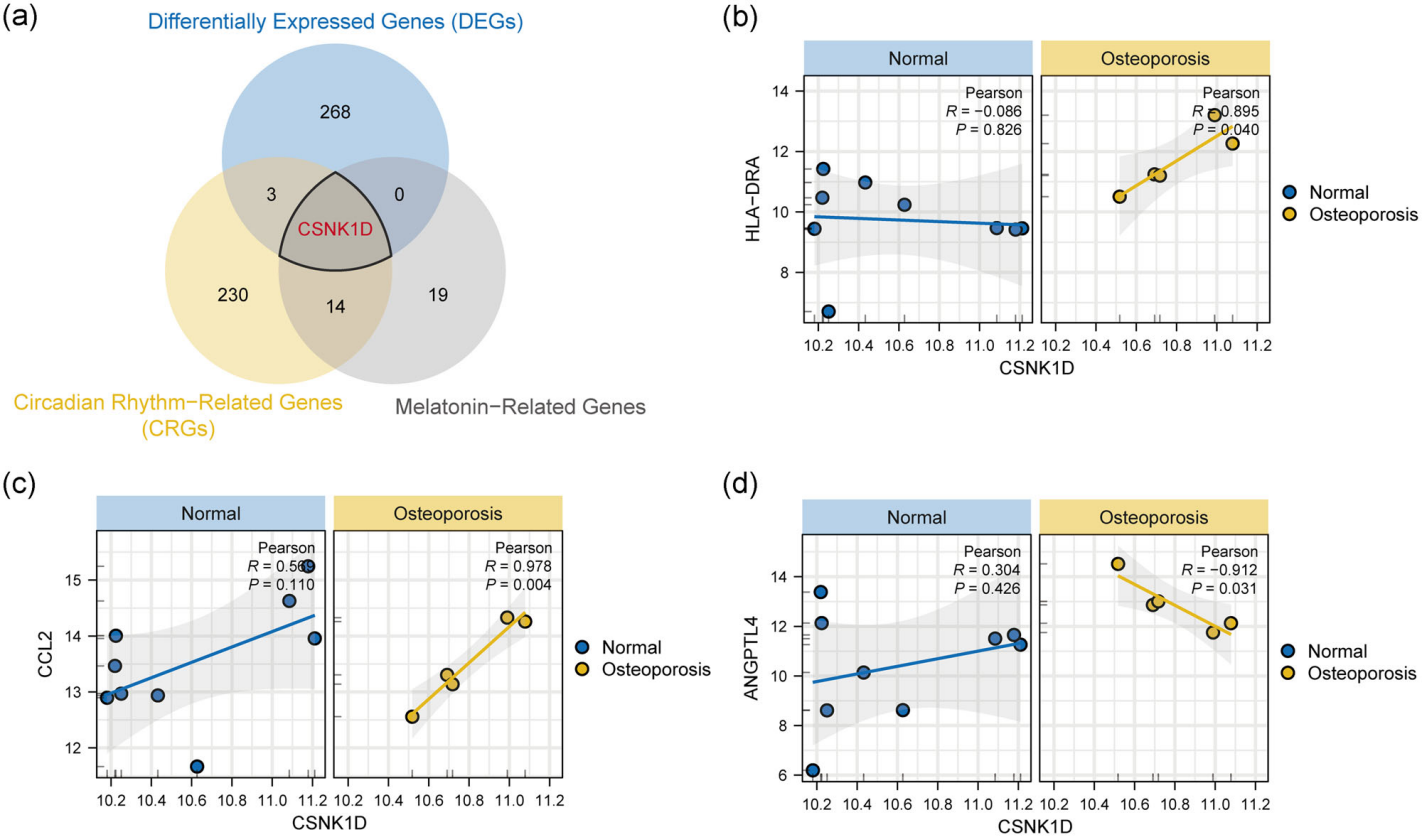
Identification of the key gene *CSNK1D* and correlation analysis. (a) Intersection of differential genes with circadian rhythm‐related genes and melatonin‐related genes. (b) Scatter plot of correlation analysis between *CSNK1D* and *HLA‐DRA*. (c) Scatter plot of correlation analysis between *CSNK1D* and *CCL2*. (d) Scatter plot of correlation analysis between *CSNK1D* and *ANGPTL4*.

Intersection of 272 DEGs, 248 circadian rhythm‐related genes and 34 melatonin‐related genes pinpointed *CSNK1D* as a pivotal gene associated with melatonin pathways (Figure 4a). Correlation analysis in OP patients showed significant positive correlations between *CSNK1D* and *HLA‐DRA* (*r* = 0.895, *P* = 0.04; Figure 4b), *CCL2* (*r* = 0.978, *P* = 0.004; Figure 4c), and negative correlations with *ANGPTL4* (*R* = −0.912, *P* = 0.031; Figure 4d).

### 3.5 Construction of PPI network and functional enrichment analysis of CSNK1D

**FIGURE 5:**
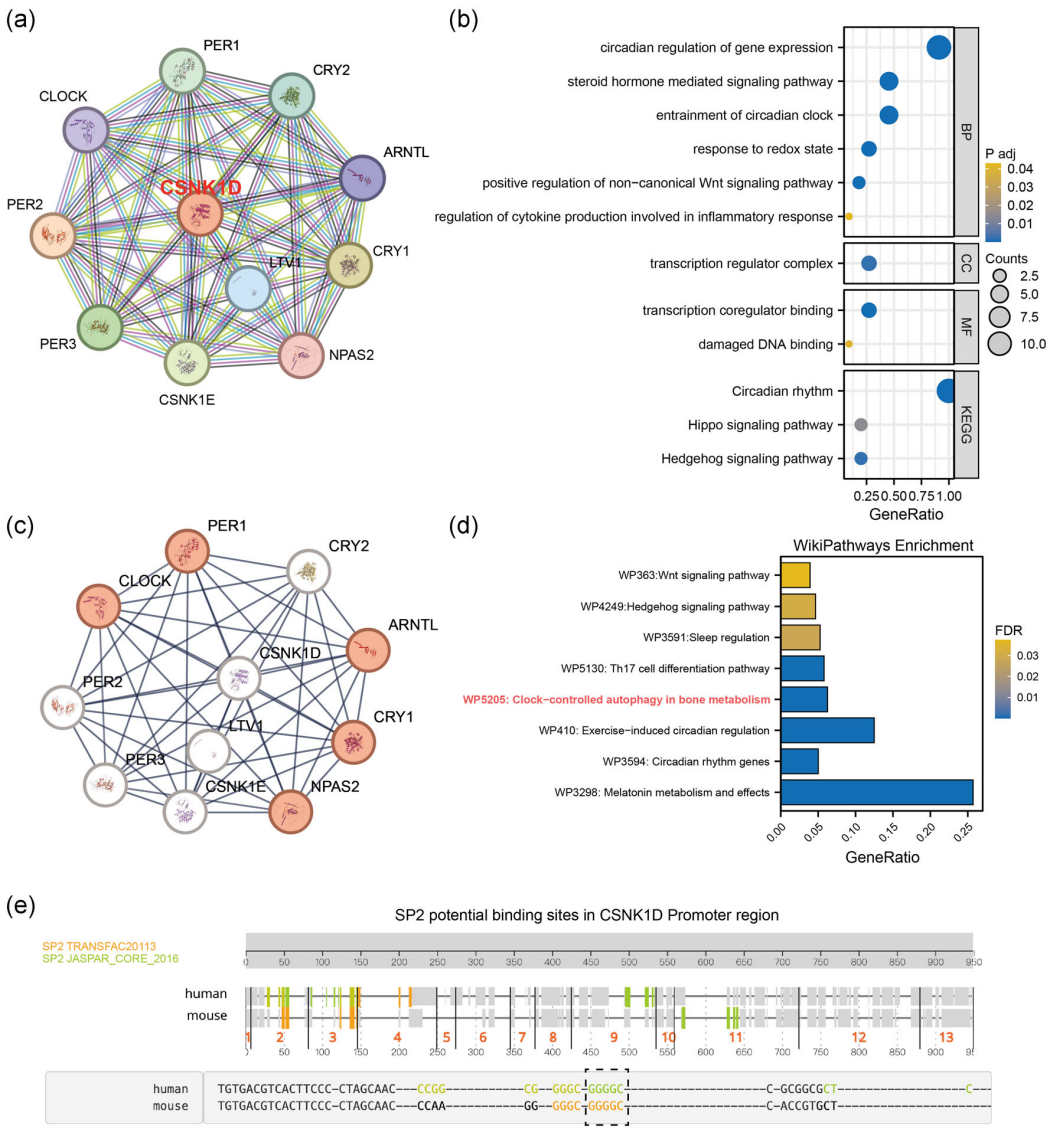
Construction of protein−protein interaction (PPI) network, functional enrichment and potential transcription factor analysis of *CSNK1D*. (a) PPI networks between *CSNK1D* and other circadian rhythm‐related proteins. (b) GO/KEGG enrichment analysis of *CSNK1D*. (c) PPI network analysis of *CSNK1D* and bone metabolic protein. (d) WikiPathways enrichment analysis of *CSNK1D*. (e) Potential transcription factor SP2 binding sites of *CSNK1D*.

We investigated the PPIs and functional enrichment of *CSNK1D* using the STRING database. The PPI network revealed key interactions between *CSNK1D* and other circadian rhythm‐related proteins (Figure 5a). Functional enrichment analysis of these interactions showed involvement in circadian gene expression regulation, Wnt signalling and inflammatory responses (Figure 5b). To explore the role of *CSNK1D* in bone metabolism, we conducted a dedicated PPI network analysis. The results demonstrated that *CSNK1D* is closely connected with key circadian rhythm‐related proteins, such as CLOCK, PER1, CRY2, ARNTL and NPAS2, all of which are implicated in bone metabolism (Figure 5c). Pathway enrichment analysis revealed that *CSNK1D* is involved in several critical pathways, including circadian regulation in bone metabolism, Wnt signalling, inflammatory responses and clock‐controlled autophagy. These findings underscore the multifaceted role of *CSNK1D* in maintaining bone health and its potential as a therapeutic target for OP (Figure 5d). Using the KnockTF database, we found that *CSNK1D* gene expression was significantly downregulated after SP2 knockdown. We then predicted the sequence of the potential SP2 binding site in the *CSNK1D* promoter region by ConTra v.3 database: ‐GGGGC‐. This binding sequence predicted binding in both human and mouse data (Figure 5e).

### 3.6 The role of *CSNK1D* in osteoclastogenesis in vitro

**FIGURE 6:**
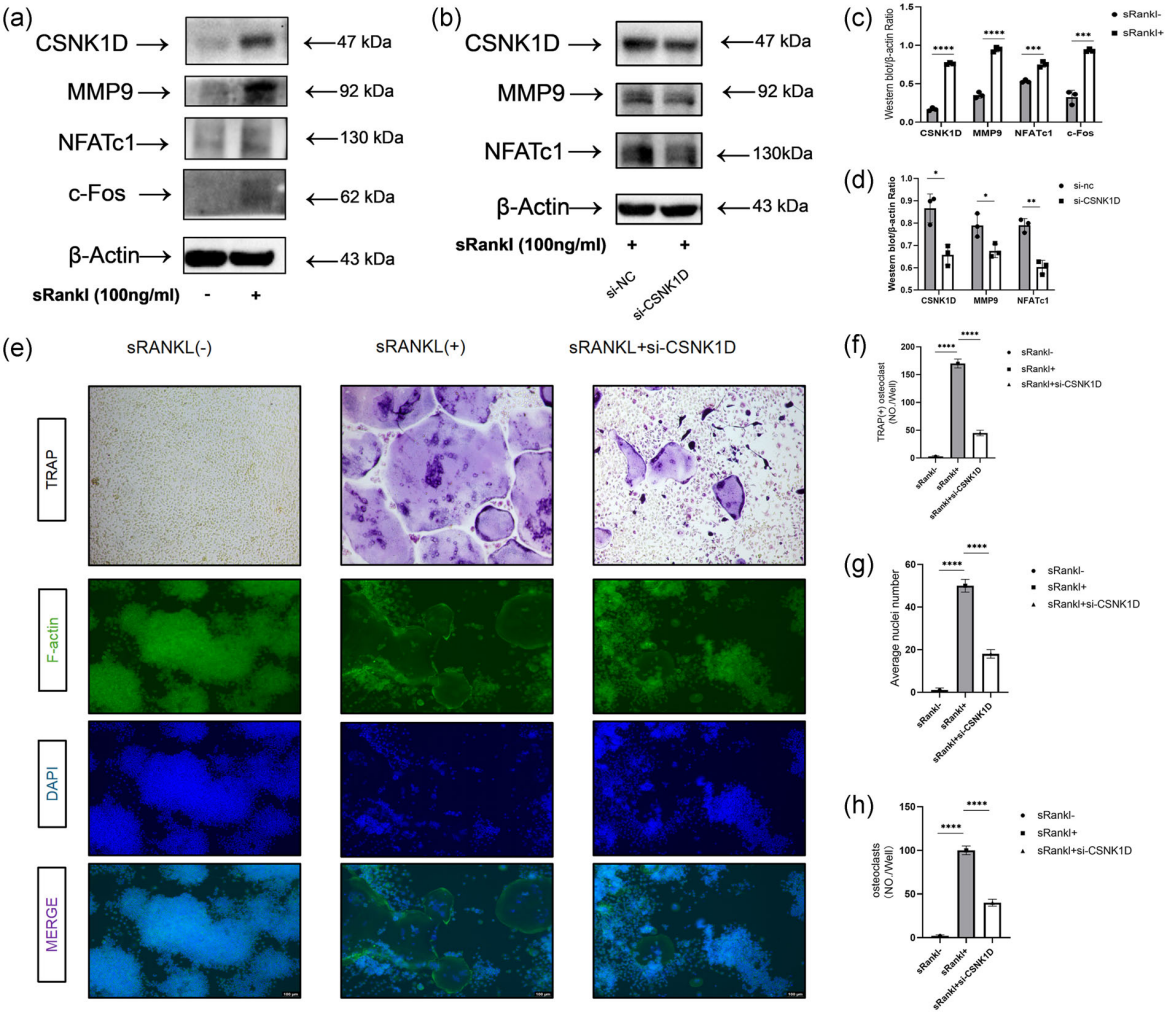
High expression of *CSNK1D* promotes osteoclast differentiation. (a) Relative expression of marker genes (*MMP9*, *NFATc1* and *c‐Fos*) and *CSNK1D* at the protein level after stimulation of RAW264.7 cells with or without RANKL for 4 days. (b) Relative expression of marker genes (*MMP9*, *NFATc1* and *c‐Fos*) and *CSNK1D* at the protein level after treatment of RANKL‐induced RAW264.7 cells with si‐NC and si‐CSNK1D, respectively, for 4 days. (c) Quantification of MMP9, NFATc1, c‐Fos and CSNK1D relative to β‐actin. (d) Quantification of MMP9, NFATc1 and CSNK1D relative to β‐actin. (e) TRAP staining and F‐actin fluorescence staining were performed on representative images of RAW264.7 cells induced for 4 days without RANKL, with RANKL or with RANKL + si‐CSNK1D. (f) Quantification of TRAP^+^ multinucleated osteoclasts (≥3 nuclei) per well. (g,h) Quantitative analysis of the number of osteoclasts and average osteoclast area. *n* = 3 per group, Data represent the mean ± SD. ^*^ *P* < 0.05, ^**^ *P *< 0.01, ^***^ *P *< 0.001 and ^****^ *P* < 0.0001 based on one‐way ANOVA. si‐NC, Negative control small interfering RNA; si‐CSNK1D, Small interfering RNA targeting the CSNK1D gene.

To validate the potential of RAW264.7 cells for osteoclastogenesis and to quantify the disparity in *CSNK1D* expression between induced and non‐induced conditions, we used western blot analysis. Our findings, revealed 4 days after induction of OCs differentiation, demonstrated a marked upregulation of both osteoclast‐specific markers and *CSNK1D* protein levels in the induction group compared with the control group devoid of RANKL treatment (CSNK1D, *P* < 0.0001; MMP9, *P* < 0.0001; NFATC1, *P* = 0.000784; C‐Fos, *P* = 0.000430; Figure 6a,c).

To elucidate the functional role of *CSNK1D* in this process, we used siRNA‐mediated knockdown in RAW264.7 cells. Four days after induction of OCs differentiation, western blot analysis confirmed significant downregulation of both OC markers and CSNK1D expression in the Small interfering RNA targeting the CSNK1D gene (si‐CSNK1D) group, in comparison to the Negative control small interfering RNA (si‐NC) group (CSNK1D, *P* = 0.010273; MMP9, *P* = 0.020647; NFATc1, *P* = 0.003213; Figure 6b,d).

Moreover, TRAP staining performed 4 days post‐induction revealed a substantial increase in the number of TRAP^+^ OCs in the sRANKL^+^ group compared with the sRANKL^−^ group (*P* < 0.0001). Notably, the number of TRAP^+^ cells in the RANKL^+^ si‐CSNK1D group was also significantly less than that in the sRANKL^+^ group (*P* < 0.0001; Figure 6e,f), indicating that *CSNK1D* silencing impairs OC differentiation. In parallel, F‐actin fluorescence staining revealed the formation of prominent F‐actin rings in the sRANKL‐induced group, characteristic of mature OCs, whereas such structures were absent in the sRANKL^−^ group. Notably, the RANKL^+^ si‐CSNK1D group exhibited a notable decrease in F‐actin ring formation compared with the sRANKL^+^ group (Figure 6e,g,h). Specifically, the number of osteoclast nuclei in the RANKL^+^ si‐CSNK1D group and the SRANKL^−^ group was significantly reduced compared with the sRANKL^+^ group (*P* < 0.0001). Meanwhile, compared with the sRANKL^+^ group, the average osteoclast areas of the sRANKL^−^ and RANKL^+^ si‐CSNK1D groups were significantly decreased (*P* < 0.0001), which confirmed the inhibitory effect of CSNK1D knockdown on OC differentiation and maturation.

In conclusion, *CSNK1D* plays a pivotal role in facilitating OC differentiation, as evidenced by its upregulation during the differentiation process and the subsequent suppression of osteoclastic markers and morphological characteristics upon its knockdown.

### 3.7 Increased expression of *CSNK1D* in ovariectomized rat femur

**FIGURE 7:**
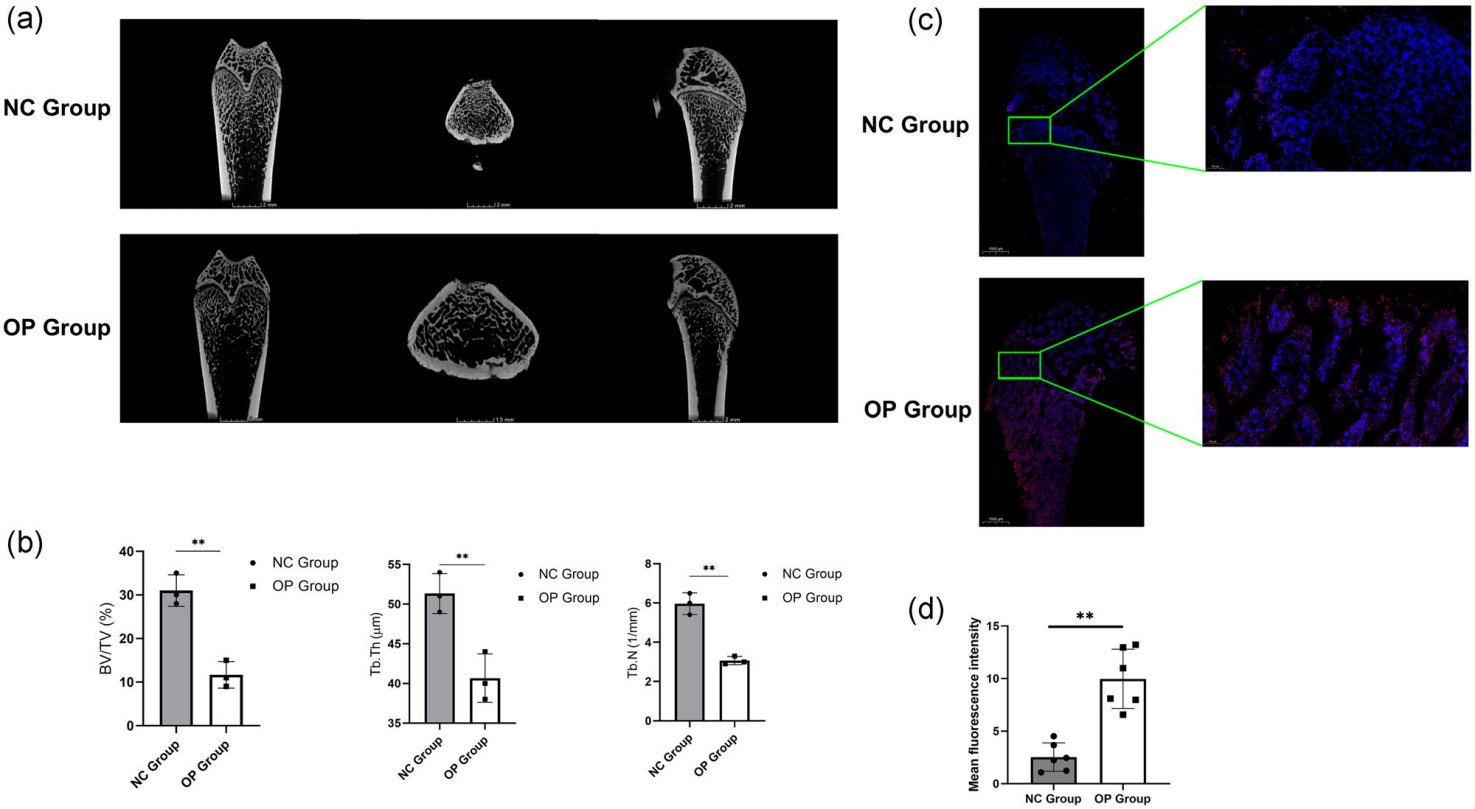
Increased expression of CSNK1D in ovariectomized rat femur. (a) Mico‐CT of femur in sham operation (NC) group and ovariectomy (OP) group. (b) Comparison of bone volume fraction (BV/TV), trabecular thickness (Tb.Th) and the number of trabeculae (Tb.N) in each group. (c) Immunofluorescence staining of femur in NC group and OP group. (d) Semi‐quantitative analysis of CSNK1D fluorescence intensity in femur sections from OP group and NC group. *n* = 6 per group; data represent the mean ± SD. ^**^ *P *< 0.01 based on one‐way ANOVA.

To investigate the in vivo expression pattern of *CSNK1D* in the context of OP, we used an ovariectomized rat model. Micro‐CT analysis revealed a significant decrease in bone mass in the OP group compared with the sham‐operated control (NC) animals, manifesting as a pronounced sparsity and disorganization of bone trabeculae (Figure 7a). Quantitative assessment confirmed these observations, with bone volume fraction, trabecular thickness and the number of trabeculae being significantly lower in the OP group compared with the normal control group (*P =* 0.002115, *P* = 0.009630 and *P* = 0.001046, respectively; Figure 7b). Collectively, these results affirm the successful establishment of an osteoporotic rat model. Subsequently, we performed immunofluorescence staining on femur samples derived from both osteoporotic rats and normal control animals (Figure 7c). Our analysis unveiled a marked upregulation of *CSNK1D* expression in the femur of OP rats compared with their normal counterparts (*P *= 0.0022; Figure 7d). This finding underscores the potential involvement of *CSNK1D* in the pathological processes underlying OP, suggesting that its upregulation might contribute to the bone loss observed in this condition.

### 3.8 Effects of *CSNK1D* on osteoblast differentiation in vitro and in vivo

**FIGURE 8:**
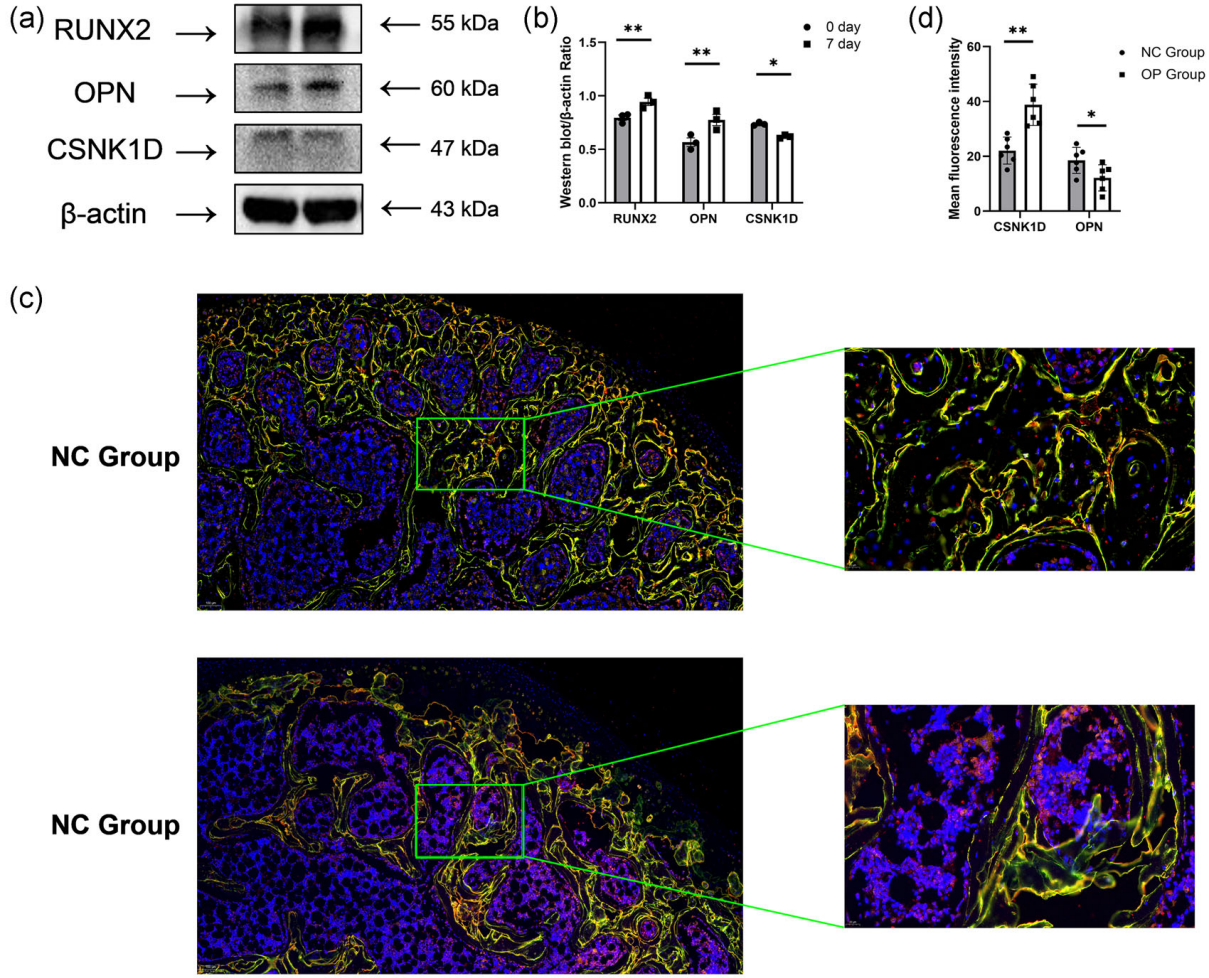
Effects of *CSNK1* *D* on osteoblast differentiation in vitro and in vivo. (a) Western blot bands showing the expression of RUNX2, OPN and CSNK1D at day 0 and day 7 of osteoblast induction. GAPDH was used as a loading control (*n* = 3 per group). (b) Quantitative analysis of RUNX2, OPN and CSNK1D expression based on band intensity (*n* = 3 per group). (c) Immunofluorescence staining of femur in sham operation (NC) group and ovariectomy (OP) group (*n* = 6 per group). (d) Semi‐quantitative analysis of CSNK1D and OPN fluorescence intensity in femur sections from the OP group and the NC group (*n* = 6 per group). Data represent the mean ± SD. ^*^ *P* < 0.05 and ^**^ *P *< 0.01 based on one‐way ANOVA.

Western blot analysis demonstrated significant changes in the expression of RUNX2, OPN and CSNK1D during osteoblast differentiation (Figure 8a). At day 7, the expression levels of RUNX2 and OPN were significantly upregulated compared with day 0 (*P *= 0.006917 and *P *= 0.001337, respectively), indicating successful induction of osteoblast differentiation. In contrast, the expression of CSNK1D was significantly decreased by day 7 (*P *= 0.015830), suggesting a potential regulatory role of CSNK1D during osteoblast differentiation (Figure 8b).

After the in vitro experiments on osteoblast differentiation, we also assessed the expression of CSNK1D and OPN in femoral head sections from the ovariectomy group and the NC group using immunofluorescence staining (Figure 8c). The results demonstrated that CSNK1D expression was significantly elevated in the OP group compared with the NC group (*P *= 0.001058). In contrast, OPN expression was markedly reduced in the OP group (*P *= 0.022193; Figure 8d). These findings align with our in vitro data, in which CSNK1D expression decreased during osteoblast differentiation, suggesting an inverse relationship between osteoblast activity and CSNK1D expression in the context of osteoporosis.

## 4 DISCUSSION

The primary aetiology of OP lies in the imbalance between bone formation and bone resorption, with a growing body of research highlighting the crucial role of OC differentiation and activation in developing this condition (Zhao, 2012). Specifically, excessive OC activity leads to accelerated bone resorption, thereby contributing significantly to OP (Kong & Penninger, 2000). Understanding the pathophysiology of OP, particularly the role of OCs and signalling pathways such as Wnt/β‐catenin, is essential for developing targeted therapies (Marini et al., 2023). Our research aims to investigate the molecular mechanisms of OP by analysing circadian rhythm and melatonin‐related genes. Using microarray data from the GSE35959 dataset, we first identified *CST3*, *RELB*, *HNRNPD* and *CSNK1D* as key hub genes related to circadian rhythm that are expressed variably in OP samples compared with controls. Secondly, we determined that *CSNK1D* is the only central gene related to circadian rhythm and melatonin that is related to OP. Finally, we verified the effects of *CSNK1D* on OCs and OP through experiments. These findings highlight *CSNK1D* as a potential new target for the treatment of OP.

Circadian rhythms, the internal 24 h cycle that governs various physiological processes, have been shown to play a crucial role in bone metabolism (Luo et al., 2021). Disruption of circadian rhythms can lead to changes in the activity of OCs and osteoblasts (Kikyo, 2024). Our study aligns with previous research demonstrating that circadian rhythm‐related genes, such as *CST3*, *RELB*, *HNRNPD* and *CSNK1D*, are associated with OP (Qin et al., 2023). For example, *CST3* is involved in bone remodelling; and Weivoda et al. (2020) showed that *LIF, CREG2, CST3, CCBE1* and *DPP4* are likely to be osteoclast‐derived coupling factors in humans. *RELB* negatively regulates bone mass as mice age and limits bone formation in healing bone defects (Yao et al., 2014). At the same time, Anastasios Fotinos et al. (2016). found that *HNRNPD* can inhibit the activity of the BMP2 3′ untranslated region in mesenchymal stem cells. In addition, these genes are also expressed functionally in OCs (Cildir et al., 2016; Wang, Qiu et al., 2020; Weivoda et al., 2020). Furthermore, many studies show that circadian rhythm genes can influence the expression of genes involved in the Wnt/β‐catenin signalling pathway, which is essential for bone formation and osteoblast differentiation (Lecarpentier et al., 2014; Vallée et al., 2018, 2022). Thus, targeting circadian rhythm disruptions might offer a new approach to preventing OP.

Melatonin is a hormone that is regulated by the circadian rhythm and has previously been linked to bone health in numerous articles. It affects bone metabolism by regulating the activity of OCs and osteoblasts (Ahmad et al., 2023). Emerging evidence also points to the involvement of melatonin in regulating the gut–bone axis, which improves OP‐related symptoms by restoring gut microbiota balance and increasing short‐chain fatty acid production (Ma et al., 2012). Ma et al. (2024) found that melatonin prevents cadmium‐induced OP by affecting the osteoblast and OC differentiation and pyroptosis in ducks. Additionally, melatonin has been shown to inhibit osteoclast activity through its free radical scavenger properties and by enhancing OPG expression, further supporting its potential in reducing bone resorption (Cardinali, 2024). Melatonin also enhances mitochondrial function in bone marrow mesenchymal stem cells, which promotes bone regeneration and improves the osteogenic capacity of these cells (Gu et al., 2024). Recent studies have revealed that melatonin promotes osteoclast apoptosis through the BMAL1/ROS/MAPK‐p38 pathway (Wang et al., 2023). Therefore, we identified *CSNK1D*, which is co‐related with circadian rhythm, melatonin and OP, as the central gene for in‐depth study.

*CSNK1D* encodes casein kinase 1δ, a protein kinase that phosphorylates key circadian regulators, affecting their stability and function (Zhang et al., 2022). The role of *CSNK1D* in OC function can be understood through its involvement in several molecular pathways. Philpott et al. (2023) demonstrated that casein kinase 1δ is essential in regulating the circadian clock by phosphorylating PER protein, which affects the stability and function of the circadian rhythm. Previous studies have shown that *CSNK1D* is a component of the Wnt/β‐catenin signalling pathway; Zhu et al. (2022) found that the circadian gene *CSNK1D* promoted the progression of hepatocellular carcinoma by activating the Wnt/β‐catenin pathway via stabilizing dishevelled segment polarity protein 3. This pathway can also inhibit OC generation by promoting the production of OPG and decreasing the expression of RANKL, thus inhibiting OC differentiation (Yang et al., 2020). This balance between OPG and RANKL is crucial for controlling OC‐mediated bone resorption. By modulating these pathways, *CSNK1D* indirectly regulates OC activity, highlighting its potential as a therapeutic target. Furthermore, *CSNK1D* could influence TNF‐α signalling (Watts et al., 1999). Given the role of TNF‐α in bone resorption, *CSNK1D* might modulate the inflammatory response in bone tissue, further supporting its dual role in regulating both circadian rhythm and bone metabolism.

Notably, recent findings suggest that the influence of melatonin on the circadian clock can also extend to the modulation of oxidative stress levels, which play a significant role in bone resorption and formation (Stacchiotti et al., 2020). This emphasizes the importance of *CSNK1D* in both circadian rhythm regulation and bone health. Finally, the interaction of *CSNK1D* with autophagy pathways presents another intriguing avenue of research (Nakatogawa, 2015). Autophagy, which is under circadian control, plays a role in OC survival and function (Wang, Xu et al., 2020). Dysregulation of autophagy owing to disrupted circadian rhythms can impair bone homeostasis, suggesting that *CSNK1D* might influence bone metabolism through the regulation of autophagic processes (Ma et al., 2012).

In addition to its role in osteoclast regulation, *CSNK1D* might also influence osteoblast activity. Osteoblasts, responsible for producing bone matrix and regulating mineralization, operate under circadian control similar to OCs (Wu et al., 2019). Evidence suggests that circadian clock genes can modulate RUNX2 expression, thereby impacting osteoblast function (Reale et al., 2013). Given that *CSNK1D* regulates the stability of circadian proteins, such as PER and CRY, it can influence the timing of RUNX2 expression through circadian modulation, which ensures optimal synchronization between bone formation and metabolic cycles (Chan et al., 2021).

Moreover, the regulatory influence of *CSNK1D* on circadian rhythms extends beyond bone‐specific pathways. Its role as a core circadian clock regulator, via the phosphorylation of PER proteins, affects systemic processes such as metabolism, sleep and inflammation (Burger et al., 2023; Kripke et al., 2015). Importantly, circadian disruptions caused by dysregulated *CSNK1D* expression or function can lead to metabolic imbalances, which have downstream effects on bone metabolism. For example, studies have shown that chronodisruption (such as shift work or irregular light exposure) leads to increased bone resorption and decreased bone formation, contributing to osteoporosis (Swanson, 2022). *CSNK1D*, as a pivotal circadian gene, might modulate these systemic metabolic effects, linking circadian health with bone health. Additionally, *CSNK1D*, by regulating circadian rhythms, might enhance the synchrony between melatonin secretion and bone remodelling processes, further emphasizing its potential as a therapeutic target for bone diseases.

We used 12‐week‐old female Sprague–Dawley rats, and although the bones of these rats were not yet fully developed, young rats are commonly used in ovariectomy‐induced osteoporosis models. This is because ovariectomy in younger rats reliably induces bone loss similar to the postmenopausal osteoporosis observed in humans (Chen et al., 2023; Guo et al., 2023; Zhan et al., 2023). Although these rats are still actively growing, we believe that the role of *CSNK1D* in bone loss remains relevant across different age groups. However, it is plausible that age‐related factors, such as skeletal maturity and bone turnover rates, might influence *CSNK1D* expression and activity in osteoclasts and osteoblasts. Future studies using older rats or alternative models that more closely resemble the aged human population would provide further insights into how *CSNK1D* functions at different life stages.

Despite these promising findings, our study has some limitations. Firstly, the study relies on microarray data from a single dataset (GSE35959), which limits the generalizability of our findings. A larger and more diverse cohort is required to validate these results. Secondly, a major limitation is the lack of in‐depth mechanistic studies on how *CSNK1D* affects OCs and osteoblasts specifically. Future studies should focus on elucidating these mechanisms through in vivo and in vitro experiments. Additionally, our study is based on microarray data, and further validation using other techniques, such as RNA sequencing or proteomics, is necessary to confirm our findings. Finally, although our study identifies key genes and pathways associated with OP, the causal relationship between circadian disruption and bone health needs further investigation.

## 5 CONCLUSION

In conclusion, this is the first study to link a circadian rhythm‐related and melatonin‐related gene, *CSNK1D*, to bone health. Our findings provide a strong foundation for future research aimed at developing targeted therapies for OP, potentially improving the quality of life for individuals affected by this debilitating condition.

## AUTHOR CONTRIBUTIONS

Conception or design of the work: Jiewen Zhang, Shaobo Wu, Kunzheng Wang, Run Tian and Pei Yang. Acquisition, analysis, or interpretation of data for the work: Jiewen Zhang, Shaobo Wu, Fangze Xing, Ning Kong, Yiwei Zhao, Xudong Duan and Yiyang Li. Drafting of the work or revising it critically for important intellectual content: Jiewen Zhang, Shaobo Wu, Fangze Xing, Ning Kong, Yiwei Zhao, Xudong Duan, Yiyang Li, Kunzheng Wang, Run Tian and Pei Yang. All authors have approved the final version of the manuscript. All authors agree to be accountable for all aspects of the work in ensuring that questions related to the accuracy or integrity of any part of the work are appropriately investigated and resolved. All persons designated as authors qualify for authorship, and all those who qualify for authorship are listed.

## CONFLICT OF INTEREST

None declared.

## Data Availability

The datasets generated and/or analysed during the present study are available in the GEO repository, https://www.ncbi.nlm.nih.gov/geo/.

## References

[eph13688-bib-0001] Ahmad, S. B. , Ali, A. , Bilal, M. , Rashid, S. M. , Wani, A. B. , Bhat, R. R. , & Rehman, M. U. (2023). Melatonin and health: Insights of melatonin action, biological functions, and associated disorders. Cellular and Molecular Neurobiology, 43(6), 2437–2458. 36752886 10.1007/s10571-023-01324-w PMC9907215

[eph13688-bib-0002] Amstrup, A. K. , Sikjaer, T. , Mosekilde, L. , & Rejnmark, L. (2013). Melatonin and the skeleton. Osteoporosis International, 24(12), 2919–2927. 23716040 10.1007/s00198-013-2404-8

[eph13688-bib-0003] Black, D. M. , & Rosen, C. J. (2016). Clinical practice. Postmenopausal osteoporosis. New England Journal of Medicine, 374(3), 254–262. 26789873 10.1056/NEJMcp1513724

[eph13688-bib-0004] Boyce, B. F. (2013). Advances in the regulation of osteoclasts and osteoclast functions. Journal of Dental Research, 92(10), 860–867. 23906603 10.1177/0022034513500306 PMC3775372

[eph13688-bib-0005] Boyce, B. F. , & Xing, L. (2007). Biology of RANK, RANKL, and osteoprotegerin. Arthritis Research & Therapy, 9(Suppl 1), S1. 17634140 10.1186/ar2165 PMC1924516

[eph13688-bib-0006] Boyce, B. F. , & Xing, L. (2008). Functions of RANKL/RANK/OPG in bone modeling and remodeling. Archives of Biochemistry and Biophysics, 473(2), 139–146. 18395508 10.1016/j.abb.2008.03.018 PMC2413418

[eph13688-bib-0007] Boyle, W. J. , Simonet, W. S. , & Lacey, D. L. (2003). Osteoclast differentiation and activation. Nature, 423(6937), 337–342. 12748652 10.1038/nature01658

[eph13688-bib-0008] Burger, K. L. , Fernandez, M. R. , Meads, M. B. , Sudalagunta, P. , Oliveira, P. S. , Renatino Canevarolo, R. , Alugubelli, R. R. , Tungsevik, A. , De Avila, G. , Silva, M. , Graeter, A. I. , Dai, H. A. , Vincelette, N. D. , Prabhu, A. , Magaletti, D. , Yang, C. , Li, W. , Kulkarni, A. , Hampton, O. , … Shain, K. H. (2023). CK1δ and CK1ε signaling sustains mitochondrial metabolism and cell survival in multiple myeloma. Cancer Research, 83(23), 3901–3919. 37702657 10.1158/0008-5472.CAN-22-2350 PMC10690099

[eph13688-bib-0009] Camacho, P. M. , Petak, S. M. , Binkley, N. , Diab, D. L. , Eldeiry, L. S. , Farooki, A. , Harris, S. T. , Hurley, D. L. , Kelly, J. , Lewiecki, E. M. , Pessah‐Pollack, R. , Mcclung, M. , Wimalawansa, S. J. , & Watts, N. B. (2020). American association of Clinical Endocrinologists/American College of Endocrinology Clinical Practice Guidelines for the diagnosis and treatment of postmenopausal osteoporosis‐2020 update. Endocrine Practice, 26, 1–46. 10.4158/GL-2020-0524SUPPL 32427503

[eph13688-bib-0010] Cardinali, D. P. (2024). Melatonin as a chronobiotic/cytoprotective agent in bone. Doses involved. Journal of Pineal Research, 76(1), e12931. 38083808 10.1111/jpi.12931

[eph13688-bib-0011] Cardinali, D. P. , Ladizesky, M. G. , Boggio, V. , Cutrera, R. A. , & Mautalen, C. (2003). Melatonin effects on bone: Experimental facts and clinical perspectives. Journal of Pineal Research, 34(2), 81–87. 12562498 10.1034/j.1600-079x.2003.00028.x

[eph13688-bib-0012] Chan, W. C. W. , Tan, Z. , To, M. K. T. , & Chan, D. (2021). Regulation and role of transcription factors in osteogenesis. International Journal of Molecular Sciences, 22(11), 5445. 34064134 10.3390/ijms22115445 PMC8196788

[eph13688-bib-0013] Chen, Z. , Lv, M. , Liang, J. , Yang, K. , Li, F. , Zhou, Z. , Qiu, M. , Chen, H. , Cai, Z. , Cui, W. , & Li, Z. (2023). Neuropeptide Y‐mediated gut microbiota alterations aggravate postmenopausal osteoporosis. Advanced Science (Weinheim), 10(33), e2303015. 10.1002/advs.202303015 PMC10667841 37857552

[eph13688-bib-0014] Cildir, G. , Low, K. C. , & Tergaonkar, V. (2016). Noncanonical NF‐κB signaling in health and disease. Trends in Molecular Medicine, 22(5), 414–429. 27068135 10.1016/j.molmed.2016.03.002

[eph13688-bib-0015] Clynes, M. A. , Harvey, N. C. , Curtis, E. M. , Fuggle, N. R. , Dennison, E. M. , & Cooper, C. (2020). The epidemiology of osteoporosis. British Medical Bulletin, 133, 105–117. 32282039 10.1093/bmb/ldaa005 PMC7115830

[eph13688-bib-0016] Coughlan, T. , & Dockery, F. (2014). Osteoporosis and fracture risk in older people. Clinical Medicine (London), 14(2), 187–191. 10.7861/clinmedicine.14-2-187 PMC4953292 24715132

[eph13688-bib-0017] Fotinos, A. , Fritz, D. T. , Lisica, S. , Liu, Y. , & Rogers, M. B. (2016). Competing repressive factors control bone morphogenetic protein 2 (BMP2) in mesenchymal cells. Journal of Cellular Biochemistry, 117(2), 439–447. 26212702 10.1002/jcb.25290 PMC4913784

[eph13688-bib-0018] Gu, C. , Zhou, Q. , Hu, X. , Ge, X. , Hou, M. , Wang, W. , Liu, H. , Shi, Q. , Xu, Y. , Zhu, X. , Yang, H. , Chen, X. , Liu, T. , & He, F. (2024). Melatonin rescues the mitochondrial function of bone marrow‐derived mesenchymal stem cells and improves the repair of osteoporotic bone defect in ovariectomized rats. Journal of Pineal Research, 76(1), e12924. 37941528 10.1111/jpi.12924

[eph13688-bib-0019] Guo, M. , Liu, H. , Yu, Y. , Zhu, X. , Xie, H. , Wei, C. , Mei, C. , Shi, Y. , Zhou, N. , Qin, K. , & Li, W. (2023). Lactobacillus rhamnosus GG ameliorates osteoporosis in ovariectomized rats by regulating the Th17/Treg balance and gut microbiota structure. Gut Microbes, 15(1), 2190304. 36941563 10.1080/19490976.2023.2190304 PMC10038048

[eph13688-bib-0020] Hadjidakis, D. J. , & Androulakis, I. I (2006). Bone remodeling. Annals of the New York Academy of Sciences, 1092(1), 385–396. 17308163 10.1196/annals.1365.035

[eph13688-bib-0021] Huybrechts, Y. , Mortier, G. , Boudin, E. , & Van Hul, W. (2020). WNT signaling and bone: Lessons from skeletal dysplasias and disorders. Frontiers in Endocrinology (Lausanne), 11, 165. 10.3389/fendo.2020.00165 PMC7160326 32328030

[eph13688-bib-0022] Juliana, N. , Azmi, L. , Effendy, N. M. , Mohd Fahmi Teng, N. I. , Abu, I. F. , Abu Bakar, N. N. , Azmani, S. , Yazit, N. A. A. , Kadiman, S. , & Das, S. (2023). Effect of circadian rhythm disturbance on the human musculoskeletal system and the importance of nutritional strategies. Nutrients, 15(3), 734. 36771440 10.3390/nu15030734 PMC9920183

[eph13688-bib-0023] Kanis, J. A. (1994). Assessment of fracture risk and its application to screening for postmenopausal osteoporosis: Synopsis of a WHO report. WHO study group. Osteoporosis International, 4(6), 368–381. 7696835 10.1007/BF01622200

[eph13688-bib-0024] Kanis, J. A. , Cooper, C. , Rizzoli, R. , & Reginster, J. Y. (2019). European guidance for the diagnosis and management of osteoporosis in postmenopausal women. Osteoporosis International, 30(1), 3–44. 30324412 10.1007/s00198-018-4704-5 PMC7026233

[eph13688-bib-0025] Kikyo, N. (2024). Circadian regulation of bone remodeling. International Journal of Molecular Sciences, 25(9), 4717. 38731934 10.3390/ijms25094717 PMC11083221

[eph13688-bib-0026] Komatsu, K. , Ideno, H. , Nakashima, K. , Udagawa, N. , Kobayashi, Y. , Kimura, H. , Tachibana, M. , Yamashita, T. , & Nifuji, A. (2024). The G9a histone methyltransferase represses osteoclastogenesis and bone resorption by regulating NFATc1 function. Federation of American Societies for Experimental Biology Journal, 38(13), e23779.38967255 10.1096/fj.202400449RR

[eph13688-bib-0027] Kong, Y. Y. , & Penninger, J. M. (2000). Molecular control of bone remodeling and osteoporosis. Experimental Gerontology, 35(8), 947–956. 11121682 10.1016/s0531-5565(00)00178-9

[eph13688-bib-0028] Kripke, D. F. , Kline, L. E. , Nievergelt, C. M. , Murray, S. S. , Shadan, F. F. , Dawson, A. , Poceta, J. S. , Cronin, J. , Jamil, S. M. , Tranah, G. J. , Loving, R. T. , Grizas, A. P. , & Hahn, E. K. (2015). Genetic variants associated with sleep disorders. Sleep Medicine, 16(2), 217–224. 25660813 10.1016/j.sleep.2014.11.003 PMC4352103

[eph13688-bib-0029] Krishnacoumar, B. , Stenzel, M. , Garibagaoglu, H. , Omata, Y. , Sworn, R. L. , Hofmann, T. , Ipseiz, N. , Czubala, M. A. , Steffen, U. , Maccataio, A. , Stoll, C. , Böhm, C. , Herrmann, M. , Uderhardt, S. , Jenkins, R. H. , Taylor, P. R. , Grüneboom, A. , Zaiss, M. M. , Schett, G. , … Scholtysek, C. (2024). Caspase‐8 promotes scramblase‐mediated phosphatidylserine exposure and fusion of osteoclast precursors. Bone Research, 12(1), 40. 38987568 10.1038/s41413-024-00338-4 PMC11237014

[eph13688-bib-0030] Lecarpentier, Y. , Claes, V. , Duthoit, G. , & Hébert, J. L. (2014). Circadian rhythms, Wnt/beta‐catenin pathway and PPAR alpha/gamma profiles in diseases with primary or secondary cardiac dysfunction. Frontiers in Physiology, 5, 429. 25414671 10.3389/fphys.2014.00429 PMC4220097

[eph13688-bib-0031] Liu, F. , Kohlmeier, S. , & Wang, C. Y. (2008). Wnt signaling and skeletal development. Cell‐Signalling, 20(6), 999–1009. 18164181 10.1016/j.cellsig.2007.11 PMC2413267

[eph13688-bib-0032] Liu, X. H. , Kirschenbaum, A. , Yao, S. , & Levine, A. C. (2007). Androgens promote preosteoblast differentiation via activation of the canonical Wnt signaling pathway. Annals of the New York Academy of Sciences, 1116(1), 423–431. 17646262 10.1196/annals.1402.017

[eph13688-bib-0033] Luo, B. , Zhou, X. , Tang, Q. , Yin, Y. , Feng, G. , Li, S. , & Chen, L. (2021). Circadian rhythms affect bone reconstruction by regulating bone energy metabolism. Journal of Translational Medicine, 19(1), 410. 34579752 10.1186/s12967-021-03068-x PMC8477514

[eph13688-bib-0034] Ma, D. , Li, S. , Molusky, M. M. , & Lin, J. D. (2012). Circadian autophagy rhythm: A link between clock and metabolism? Trends in Endocrinology and Metabolism, 23(7), 319–325. 22520961 10.1016/j.tem.2012.03.004 PMC3389582

[eph13688-bib-0035] Ma, Y. , Su, Q. , Zhao, L. , Zhu, J. , Zhao, H. , Song, R. , Zou, H. , & Liu, Z. (2024). Melatonin prevents cadmium‐induced osteoporosis by affecting the osteoblast and osteoclast differentiation and pyroptosis in duck. Poultry Science, 103(9), 103934. 10.1016/j.psj.2024.103934 PMC11294718 38981361

[eph13688-bib-0036] Macdonald, I. J. , Tsai, H. C. , Chang, A. C. , Huang, C. C. , Yang, S. F. , & Tang, C. H. (2021). Melatonin inhibits osteoclastogenesis and osteolytic bone metastasis: Implications for osteoporosis. International Journal of Molecular Sciences, 22(17), 9435. 34502344 10.3390/ijms22179435 PMC8430520

[eph13688-bib-0037] Maeda, K. , Kobayashi, Y. , Udagawa, N. , Uehara, S. , Ishihara, A. , Mizoguchi, T. , Kikuchi, Y. , Takada, I. , Kato, S. , Kani, S. , Nishita, M. , Marumo, K. , Martin, T. J. , Minami, Y. , & Takahashi, N. (2012). Wnt5a‐Ror2 signaling between osteoblast‐lineage cells and osteoclast precursors enhances osteoclastogenesis. Nature Medicine, 18(3), 405–412. 10.1038/nm.2653 22344299

[eph13688-bib-0038] Manolagas, S. C. , & Jilka, R. L. (1995). Bone marrow, cytokines, and bone remodeling. Emerging insights into the pathophysiology of osteoporosis. New England Journal of Medicine, 332(5), 305–311. 7816067 10.1056/NEJM199502023320506

[eph13688-bib-0039] Marini, F. , Giusti, F. , Palmini, G. , & Brandi, M. L. (2023). Role of Wnt signaling and sclerostin in bone and as therapeutic targets in skeletal disorders. Osteoporosis International, 34(2), 213–238. 35982318 10.1007/s00198-022-06523-7

[eph13688-bib-0040] Mcclung, M. R. (1999). Therapy for fracture prevention. Journal of the American Medical Association, 282(7), 687–9. 10517723 10.1001/jama.282.7.687

[eph13688-bib-0041] Nakatogawa, H. (2015). Hrr25: An emerging major player in selective autophagy regulation in Saccharomyces cerevisiae. Autophagy, 11(2), 432–433. 25700828 10.1080/15548627.2015.1017195 PMC4502746

[eph13688-bib-0042] Neer, R. M. , Arnaud, C. D. , Zanchetta, J. R. , Prince, R. , Gaich, G. A. , Reginster, J. Y. , Hodsman, A. B. , Eriksen, E. F. , Ish‐Shalom, S. , Genant, H. K. , Wang, O. , & Mitlak, B. H. (2001). Effect of parathyroid hormone (1‐34) on fractures and bone mineral density in postmenopausal women with osteoporosis. New England Journal of Medicine, 344(19), 1434–1441. 11346808 10.1056/NEJM200105103441904

[eph13688-bib-0043] Philpott, J. M. , Freeberg, A. M. , Park, J. , Lee, K. , Ricci, C. G. , Hunt, S. R. , Narasimamurthy, R. , Segal, D. H. , Robles, R. , Cai, Y. , Tripathi, S. , Mccammon, J. A. , Virshup, D. M. , Chiu, J. C. , Lee, C. , & Partch, C. L. (2023). PERIOD phosphorylation leads to feedback inhibition of CK1 activity to control circadian period. Molecular Cell, 83(10), 1677–1692.e8.e8. 37207626 10.1016/j.molcel.2023.04.019 PMC11684667

[eph13688-bib-0044] Ping, Z. , Wang, Z. , Shi, J. , Wang, L. , Guo, X. , Zhou, W. , Hu, X. , Wu, X. , Liu, Y. , Zhang, W. , Yang, H. , Xu, Y. , Gu, Y. , & Geng, D. (2017). Inhibitory effects of melatonin on titanium particle‐induced inflammatory bone resorption and osteoclastogenesis via suppression of NF‐κB signaling. Acta Biomaterialia, 62, 362–371. 28867647 10.1016/j.actbio.2017.08.046

[eph13688-bib-0045] Qin, Y. , Chen, Z. H. , Wu, J. J. , Zhang, Z. Y. , Yuan, Z. D. , Guo, D. Y. , Chen, M. N. , Li, X. , & Yuan, F. L. (2023). Circadian clock genes as promising therapeutic targets for bone loss. Biomedicine & Pharmacotherapy, 157, 114019. 36423544 10.1016/j.biopha.2022.114019

[eph13688-bib-0046] Rachner, T. D. , Khosla, S. , & Hofbauer, L. C. (2011). Osteoporosis: Now and the future. The Lancet, 377(9773), 1276–1287. 10.1016/S0140-6736(10)62349-5 PMC3555696 21450337

[eph13688-bib-0047] Reale, M. E. , Webb, I. C. , Wang, X. , Baltazar, R. M. , Coolen, L. M. , & Lehman, M. N. (2013). The transcription factor Runx2 is under circadian control in the suprachiasmatic nucleus and functions in the control of rhythmic behavior. PLoS ONE, 8(1), e54317. 23372705 10.1371/journal.pone.0054317 PMC3555987

[eph13688-bib-0048] Roodman, G. D. (1996). Advances in bone biology: The osteoclast. Endocrine Reviews, 17, 308–32. 8854048 10.1210/edrv-17-4-308

[eph13688-bib-0049] Siris, E. S. , Miller, P. D. , Barrett‐Connor, E. , Faulkner, K. G. , Wehren, L. E. , Abbott, T. A. , Berger, M. L. , Santora, A. C. , & Sherwood, L. M. (2001). Identification and fracture outcomes of undiagnosed low bone mineral density in postmenopausal women: Results from the National Osteoporosis Risk Assessment. Journal of the American Medical Association, 286(22), 2815–22. 11735756 10.1001/jama.286.22.2815

[eph13688-bib-0050] Stacchiotti, A. , Favero, G. , & Rodella, L. F. (2020). Impact of melatonin on skeletal muscle and exercise. Cells, 9(2), 288. 31991655 10.3390/cells9020288 PMC7072499

[eph13688-bib-0051] Swanson, C. (2022). Sleep disruption and bone health. Current Osteoporosis Report, 20(3), 202–212. 10.1007/s11914-022-00733-y PMC10108658 35488985

[eph13688-bib-0052] Swanson, C. M. , Kohrt, W. M. , Buxton, O. M. , Everson, C. A. , Wright, K. P., Jr. , Orwoll, E. S. , & Shea, S. A. (2018). The importance of the circadian system & sleep for bone health. Metabolism, 84, 28–43. 29229227 10.1016/j.metabol.2017.12.002 PMC5994176

[eph13688-bib-0053] Takarada, T. , Xu, C. , Ochi, H. , Nakazato, R. , Yamada, D. , Nakamura, S. , Kodama, A. , Shimba, S. , Mieda, M. , Fukasawa, K. , Ozaki, K. , Iezaki, T. , Fujikawa, K. , Yoneda, Y. , Numano, R. , Hida, A. , Tei, H. , Takeda, S. , & Hinoi, E. (2017). Bone resorption is regulated by circadian clock in osteoblasts. Journal of Bone and Mineral Research, 32(4), 872–881. 27925286 10.1002/jbmr.3053

[eph13688-bib-0054] Teitelbaum, S. L. , & Ross, F. P. (2003). Genetic regulation of osteoclast development and function. Nature Reviews Genetics, 4(8), 638–649. 10.1038/nrg1122 12897775

[eph13688-bib-0055] Titorencu, I. , Pruna, V. , Jinga, V. V. , & Simionescu, M. (2014). Osteoblast ontogeny and implications for bone pathology: An overview. Cell and Tissue Research, 355(1), 23–33. 24292720 10.1007/s00441-013-1750-3

[eph13688-bib-0056] Vallée, A. , Lecarpentier, Y. , Guillevin, R. , & Vallée, J. N. (2018). Thermodynamics in neurodegenerative diseases: Interplay between canonical WNT/beta‐catenin pathway‐PPAR gamma, energy metabolism and circadian rhythms. Neuromolecular Medicine, 20(2), 174–204. 29572723 10.1007/s12017-018-8486-x

[eph13688-bib-0057] Vallee, A. , Lecarpentier, Y. , & Vallée, J. N. (2022). WNT/β‐catenin pathway and circadian rhythms in obsessive‐compulsive disorder. Neural Regeneration Research, 17(10), 2126–2130. 35259818 10.4103/1673-5374.332133 PMC9083179

[eph13688-bib-0058] Vasey, C. , Mcbride, J. , & Penta, K. (2021). Circadian rhythm dysregulation and restoration: The role of melatonin. Nutrients, 13(10), 3480. 34684482 10.3390/nu13103480 PMC8538349

[eph13688-bib-0059] Wang, X. , Jiang, W. , Pan, K. , Tao, L. , & Zhu, Y. (2023). Melatonin induces RAW264.7 cell apoptosis via the BMAL1/ROS/MAPK‐p38 pathway to improve postmenopausal osteoporosis. Bone Joint Research, 12(11), 677–690. 37907083 10.1302/2046-3758.1211.BJR-2022-0425.R3 PMC10618049

[eph13688-bib-0060] Wang, X. , Xu, Z. , Cai, Y. , Zeng, S. , Peng, B. , Ren, X. , Yan, Y. , & Gong, Z. (2020). Rheostatic balance of circadian rhythm and autophagy in metabolism and disease. Frontiers in Cell and Developmental Biology, 8, 616434. 33330516 10.3389/fcell.2020.616434 PMC7732583

[eph13688-bib-0061] Wang, Z. , Qiu, H. , He, J. , Liu, L. , Xue, W. , Fox, A. , Tickner, J. , & Xu, J. (2020). The emerging roles of hnRNPK. Journal of Cellular Physiology, 235(3), 1995–2008. 31538344 10.1002/jcp.29186

[eph13688-bib-0062] Watts, A. D. , Hunt, N. H. , Wanigasekara, Y. , Bloomfield, G. , Wallach, D. , Roufogalis, B. D. , & Chaudhri, G. (1999). A casein kinase I motif present in the cytoplasmic domain of members of the tumour necrosis factor ligand family is implicated in ‘reverse signalling’. European Molecular Biology Organization Journal, 18(8), 2119–2126. 10.1093/emboj/18.8.2119 PMC1171296 10205166

[eph13688-bib-0063] Weivoda, M. M. , Chew, C. K. , Monroe, D. G. , Farr, J. N. , Atkinson, E. J. , Geske, J. R. , Eckhardt, B. , Thicke, B. , Ruan, M. , Tweed, A. J. , Mccready, L. K. , Rizza, R. A. , Matveyenko, A. , Kassem, M. , Andersen, T. L. , Vella, A. , Drake, M. T. , Clarke, B. L. , Oursler, M. J. , & Khosla, S. (2020). Identification of osteoclast‐osteoblast coupling factors in humans reveals links between bone and energy metabolism. Nature Communications, 11(1), 87. 10.1038/s41467-019-14003-6 PMC6946812 31911667

[eph13688-bib-0064] Weivoda, M. M. , Ruan, M. , Hachfeld, C. M. , Pederson, L. , Howe, A. , Davey, R. A. , Zajac, J. D. , Kobayashi, Y. , Williams, B. O. , Westendorf, J. J. , Khosla, S. , & Oursler, M. J. (2016). Wnt signaling inhibits osteoclast differentiation by activating canonical and noncanonical cAMP/PKA pathways. Journal of Bone and Mineral Research, 31(1), 65–75. 26189772 10.1002/jbmr.2599 PMC4758681

[eph13688-bib-0065] Wirz‐Justice, A. , Skene, D. J. , & Münch, M. (2021). The relevance of daylight for humans. Biochemical Pharmacology, 191, 114304. 33129807 10.1016/j.bcp.2020.114304

[eph13688-bib-0066] Wu, Q. Y. , Wang, J. , Tong, X. , Chen, J. , Wang, B. , Miao, Z. N. , Li, X. , Ye, J. X. , & Yuan, F. L. (2019). Emerging role of circadian rhythm in bone remodeling. Journal of Molecular Medicine (Berlin), 97(1), 19–24. 10.1007/s00109-018-1723-9 30446776

[eph13688-bib-0067] Yang, B. , Li, S. , Chen, Z. , Feng, F. , He, L. , Liu, B. , He, T. , Wang, X. , Chen, R. , Chen, Z. , Xie, P. , & Rong, L. (2020). Amyloid β peptide promotes bone formation by regulating Wnt/β‐catenin signaling and the OPG/RANKL/RANK system. Federation of American Societies for Experimental Biology Journal, 34(3), 3583–3593. 31944393 10.1096/fj.201901550R

[eph13688-bib-0068] Yao, Z. , Li, Y. , Yin, X. , Dong, Y. , Xing, L. , & Boyce, B. F. (2014). NF‐κB RelB negatively regulates osteoblast differentiation and bone formation. Journal of Bone and Mineral Research, 29(4), 866–877. 24115294 10.1002/jbmr.2108 PMC3961566

[eph13688-bib-0069] Yue, Z. , Niu, X. , Yuan, Z. , Qin, Q. , Jiang, W. , He, L. , Gao, J. , Ding, Y. , Liu, Y. , Xu, Z. , Li, Z. , Yang, Z. , Li, R. , Xue, X. , Gao, Y. , Yue, F. , Zhang, X. H. , Hu, G. , Wang, Y. , … Luo, J. (2022). RSPO2 and RANKL signal through LGR4 to regulate osteoclastic premetastatic niche formation and bone metastasis. Journal of Clinical Investigation, 132(2), e144579. 34847079 10.1172/JCI144579 PMC8759794

[eph13688-bib-0070] Zaidi, M. , Alam, A. S. , Shankar, V. S. , Bax, B. E. , Bax, C. M. , Moonga, B. S. , Bevis, P. J. , Stevens, C. , Blake, D. R. , & Pazianas, M. (1993). Cellular biology of bone resorption. Biological Reviews of the Cambridge Philosophical Society, 68(2), 197–264. 8504194 10.1111/j.1469-185x.1993.tb00996.x

[eph13688-bib-0071] Zeng, X. Z. , He, L. G. , Wang, S. , Wang, K. , Zhang, Y. Y. , Tao, L. , Li, X. J. , & Liu, S. W. (2016). Aconine inhibits RANKL‐induced osteoclast differentiation in RAW264.7 cells by suppressing NF‐κB and NFATc1 activation and DC‐STAMP expression. Acta Pharmacologica Sinica, 37(2), 255–263. 26592521 10.1038/aps.2015.85 PMC4753374

[eph13688-bib-0072] Zhan, W. , Deng, M. , Huang, X. , Xie, D. , Gao, X. , Chen, J. , Shi, Z. , Lu, J. , Lin, H. , & Li, P. (2023). Pueraria lobata‐derived exosome‐like nanovesicles alleviate osteoporosis by enhacning autophagy. Journal of Controlled Release, 364, 644–653. 37967723 10.1016/j.jconrel.2023.11.020

[eph13688-bib-0073] Zhang, Y. , Cheng, L. , Liu, Y. , Zhang, R. , Wu, Z. , Cheng, K. , & Zhang, X. (2022). Omics analyses of intestinal microbiota and hypothalamus clock genes in circadian disturbance model mice fed with green tea polyphenols. Journal of Agricultural and Food Chemistry, 70(6), 1890–1901. 35112849 10.1021/acs.jafc.1c07594

[eph13688-bib-0074] Zhao, R. (2012). Immune regulation of osteoclast function in postmenopausal osteoporosis: A critical interdisciplinary perspective. International Journal of Medical Sciences, 9(9), 825–832. 23136547 10.7150/ijms.5180 PMC3491443

[eph13688-bib-0075] Zhao, Z. , Du, Y. , Yan, K. , Zhang, L. , & Guo, Q. (2024). Exercise and osteoimmunology in bone remodeling. Federation of American Societies for Experimental Biology Journal, 38(7), e23554. 38588175 10.1096/fj.202301508RRR

[eph13688-bib-0076] Zheng, S. , Zhou, C. , Yang, H. , Li, J. , Feng, Z. , Liao, L. , & Li, Y. (2022). Melatonin accelerates osteoporotic bone defect repair by promoting osteogenesis‐angiogenesis coupling. Frontiers in Endocrinology (Lausanne), 13, 826660. 10.3389/fendo.2022.826660 PMC8902312 35273570

[eph13688-bib-0077] Zhu, M. , Zhang, J. , Bian, S. , Zhang, X. , Shen, Y. , Ni, Z. , Xu, S. , Cheng, C. , & Zheng, W. (2022). Circadian gene CSNK1D promoted the progression of hepatocellular carcinoma by activating Wnt/β‐catenin pathway via stabilizing dishevelled segment polarity protein 3. Biological Procedures Online, 24(1), 21. 36460966 10.1186/s12575-022-00183-x PMC9717411

